# Trends in Treatment of Colorectal Cancer and Short-term Outcomes During the First Wave of the COVID-19 Pandemic in Sweden

**DOI:** 10.1001/jamanetworkopen.2022.11065

**Published:** 2022-05-09

**Authors:** Karolina Eklöv, Jonas Nygren, Sven Bringman, Jenny Löfgren, Annika Sjövall, Caroline Nordenvall, Åsa H. Everhov

**Affiliations:** 1 Department of Clinical Science and Education, Södersjukhuset, Karolinska Institutet, Stockholm, Sweden; 2 Department of Surgery, Ersta Hospital, Department of Clinical Sciences, Danderyd Hospital, Karolinska Institutet, Stockholm, Sweden; 3 Department of Surgery, Södertälje Hospital, Södertälje, Department of Clinical Science and Education, Södersjukhuset, Karolinska Institutet, Stockholm, Sweden; 4 Department of Molecular Medicine and Surgery, Karolinska Institutet, Stockholm, Sweden; 5 Department of Pelvic Cancer, Gastrointestinal Oncology and Colorectal Surgery Unit, Karolinska University Hospital, Stockholm, Sweden; 6 Clinical Epidemiology Unit, Department of Medicine Solna, Karolinska Institutet, Stockholm, Sweden

## Abstract

**Importance:**

The COVID-19 pandemic has had a large impact on health care systems, not least the treatment of malignant diseases, including colorectal cancer.

**Objective:**

To investigate the treatment of colorectal cancer and short-term outcomes during the first wave of the COVID-19 pandemic, compared with the year before.

**Design, Setting, and Participants:**

This register-based cohort study used information from the Swedish Colorectal Cancer Registry during the years 2020 and 2019. Patients were from the Stockholm-Gotland region, 1 of 6 health care regions in Sweden, with approximately one-fifth of the country’s population and 8 hospitals. All patients with a diagnosis of colorectal cancer from March 1 to August 31, 2019, and March 1 to August 31, 2020, were eligible. Data were analyzed from May to June 2021.

**Exposures:**

Diagnosis of colorectal cancer during the peak of the COVID-19 pandemic in 2020.

**Main Outcomes and Measures:**

The study aimed to compare the number of patients, time to surgery, operation methods, short-term complications, and residents’ involvement in surgical practice between 2019 and 2020. Subanalyses were conducted for colon and rectal cancer.

**Results:**

A total of 1140 patients (583 men [51%]; median [IQR] age, 74 [26-99] years in 2019 and 73 [24-96] years in 2020) were enrolled. Fewer patients received a diagnosis of colorectal cancer in March through August 2020 compared with the same months in 2019 (550 vs 590 patients). Overall, patient characteristics were similar, but pretherapeutic tumor stage was more advanced in 2020 compared with 2019, with an increased proportion of T4 tumors (30% [172 patients] vs 22% [132 patients]; χ^2^_3_ = 21.1; *P* < .001). The proportion of patients undergoing laparoscopic surgery, time to surgery, and 30-day complications were similar, but the proportion of patients treated with ostomy almost doubled between 2019 and 2020, from 17% (53 patients) to 30% (96 patients) (absolute risk, 13.0%; 95% CI, 6.8% to 20.0%). Residents participated in fewer resections in 2020 than in 2019 (35% [108 patients] vs 27% [83 patients]; absolute risk, −7.90%; 95% CI, −15.00% to −0.55%). On the other hand, the treatment and outcomes for rectal cancer were comparable between the years. Significantly more patients were transferred to the nonemergency, COVID-free hospital in the region in 2020.

**Conclusions and Relevance:**

In this Swedish register-based cohort study of patients who received a diagnosis of colorectal cancer during the most intense period of the COVID-19 pandemic, a significant increase in ostomy formation for patients with colon cancer and a lower participation of residents during surgery were observed. These changes most likely were aimed at reducing complications and intensive care unit care.

## Key Points

**Question:**

Did the treatment of colorectal cancer in Stockholm, Sweden, change during the COVID-19 pandemic?

**Findings:**

In this cohort study of 1140 patients with colorectal cancer from the Swedish Colorectal Cancer Registry, the proportion of patients with colon cancer treated with ostomy almost doubled, from 17% in March to August 2019 to 30% in March to August 2020; short-term complications and time to surgery remained unchanged.

**Meaning:**

These findings suggest that changes in surgical behavior occurred during the COVID-19 pandemic, most likely aiming to reduce complications and intensive care unit care.

## Teaser

This cohort study investigates changes in the treatment of colorectal cancer (CRC) and short-term outcomes during the first wave of the COVID-19 pandemic in Sweden, compared with the year before.

## Introduction

In January 2020, the World Health Organization declared the COVID-19 pandemic, which is caused by SARS-CoV-2, to be a Public Health Emergency of International Concern.^1^ Health care systems worldwide were overwhelmed because of the prioritization of COVID-19 treatment over other diseases, and the fear of COVID-19 transmission in the general population prevented care seeking. Consequently, the pandemic affected the care of other diseases, including malignant diseases such as colorectal cancer, the fourth most common cancer worldwide.^2,3^

In a survey including surgeons from 46 countries, a clear decrease in colorectal cancer service in May and June 2020 compared with the year before was described, with reduced numbers of diagnostic endoscopies and increased use of neoadjuvant treatment in hospitals where surgery was suspended.^4^ Several countries recommended a modified surgical approach during the first stage of the pandemic in 2020, with the aim of decreasing workload in the intensive care units (ICUs) through avoidance of complications.^4,5^ According to another survey^3^ among surgeons worldwide, 96% reported that COVID-19 affected their practice, and 61% were prepared to postpone elective colorectal cancer surgery.

In Sweden, with Stockholm as the most afflicted area, the first wave of the COVID-19 pandemic peaked in April to May 2020. Screening for colorectal cancer with stool test (fecal occult blood test)^6^ was canceled from May to August,^7^ and surgery for premalignant and benign conditions was postponed. Temporary guidelines for colorectal cancer surgery were established April 3, 2020, and addressed prioritization strategies for 3 different scenarios of escalating COVID-19.^8^

The objective of this cohort study was to evaluate short-term outcomes for patients with colorectal cancer in the Stockholm-Gotland region during March to August 2020. The hypothesis was based on the assumption that fewer patients had received a diagnosis and treatment, that the time to surgery had increased, and that proportionally fewer cancer resections had been performed compared with March to August 2019.

## Methods

### Study Design

In this observational, population-based cohort study, designed in compliance with the Strengthening the Reporting of Observational Studies in Epidemiology (STROBE) reporting guideline,^9^ the surgical treatment of colorectal cancer in March to August 2020 (ie, the first most intense period of COVID-19) in Stockholm, Sweden, was compared with the corresponding 6-month period in 2019. The study was approved by the regional ethics committee in Stockholm. Informed consent was waived by the committee because the study was strictly register based.

### Setting

The Stockholm-Gotland region is 1 of 6 health care regions in Sweden. The catchment area has close to 2.5 million inhabitants (approximately one-fifth of the country’s population)^10^ and 8 hospitals: Karolinska University Hospital, South General Hospital, Danderyd Hospital, Ersta Hospital, St Göran Hospital, Södertälje Hospital, Norrtälje Hospital, and Visby Hospital. The surgical treatment of colorectal cancer is centralized to 5 of the hospitals. Ersta hospital has no emergency ward and during this period was given a role as a COVID-19–free institution with an increased capacity to manage colorectal cancer resections. All other hospitals were in an emergency state during the study period because of the large number of hospitalized patients with COVID-19.^11^ The surgeons performing colorectal cancer operations were certified specialists in coloproctology, general surgeons, and residents.

### Data Sources and Participants

The Swedish Colorectal Cancer Registry was used as data source. This register was established in 1995 for rectal cancer and in 2007 for colon cancer. It holds information on patient characteristics, preoperative and postoperative tumor staging, tumor location, surgical and oncological treatment, and short-term and long-term outcomes, including recurrence and death.^12^ The registry has a coverage of 98.8%.^13^ Data were extracted May 20, 2021. At that time, according to the registry holder, 97% of the surgical treatments for 2020 and 98% of treatments for 2019 were registered.^14^ All patients living in the Stockholm-Gotland region were included.

### Inclusion Criteria

The patients who had received a diagnosis of colorectal cancer in 2020 and 2019 in the Stockholm-Gotland region were included. The main analyses were restricted to patients who received a diagnosis from March 1 to August 31, 2019, and the same months in 2020. All patients in the Stockholm region in the actual period were included.

### Variables

Baseline characteristics included age, sex, tumor location, and tumor stage. Treatment data included discussion in pretherapeutic multidisciplinary team assessment, operating hospital, number of patients planned to undergo surgery, preoperative oncological treatment, type of surgical intervention, level of surgical expertise, acute vs elective surgery, ostomy formation, time to surgery, hospital stay, 30-day complications (defined as Clavien-Dindo score ≥2), use of ICU care, and mortality. Misclassification due to registration errors and underreporting were assumed to be similar between the 2 years.

### Statistical Analysis

Categorial variables are presented as numbers and percentages. Continuous variables are presented as median (IQR). Because each tumor is reported separately and some patients had 2 tumors, results are reported with the total number of tumors, number of patients, or number of surgical procedures as denominator. Categorial variables were compared using χ^2^ test, and continuous variables were compared with the Wilcoxon signed-rank test. Two-sided *P* < .05 denoted significance. Surgical procedures and 30-day outcomes are presented stratified for colon and rectal cancer, and differences between 2019 and 2020 are presented as absolute risk differences or mean differences with 95% CIs. The total number of ostomies was obtained by summing ostomies done as the only procedure, and those performed before and during resection surgery. In estimations of proportion of patients treated with stoma during resection surgery for colon cancer, patients were stratified for age, American Society of Anesthesiologists classification, M stage, tumor location, and emergent vs elective surgery. Results are presented as absolute risk differences between the years 2019 and 2020 in each stratum. Analyses were performed with an available case strategy; no data were imputed. Data were analyzed with statistical software from SAS version 9.4; SAS Institute) and SPSS Statistics version 26.0 (IBM Corp). Data were analyzed from May to June 2021.

## Results

A total of 1140 patients were enrolled (583 men [51%]; median [IQR] age, 74 [26-99] years in 2019 and 73 [24-96] years in 2020). The numbers of patients receiving a diagnosis of colorectal cancer were 550 in March to August 2020 and 590 in March to August 2019 (difference, 40 cases [6.7%]), with similar distributions of sex, age, and colon vs rectal cancer. Tumor stage differed between years with more advanced T stage disease in 2020 compared with 2019 (T1-T2, 22% [124 patients] vs 27% [163 patients]; T3, 33% [189 patients] vs 41% [252 patients]; and T4: 30% [172 patients] vs 22% [132 patients]; χ^2^_3_ = 21.1; *P* < .001). Fewer patients with stage N0 disease were observed in 2020 compared with 2019 (41% [235 patients] vs 46% [278 patients]; χ^2^_2_ = 7.5; *P* = .02); this difference did not correspond with a higher proportion of patients with N1-2 disease, but there was an increase in patients with stage Nx disease. There was no difference in the proportion of patients with metastases at diagnosis (21% both years; 117 patients in 2020 and 127 patients in 2019). The same proportion of patients were discussed at a pretherapeutic multidisciplinary team assessment both years (92%; 523 patients in 2020 and 560 patients in 2019). The proportions of patients with plans for surgery were comparable in 2020 and 2019 (478 patients [84%] vs 490 patients [81%]), as were patients undergoing any kind of surgical intervention (439 patients [77%] vs 444 patients [73%]) (Table 1).

**Table 1.** Characteristics of Patients Who Received Diagnoses of Colorectal Cancer in the Stockholm-Gotland Region, March 1 to August 31, 2020 and 2019

Characteristic	Patients, No. (%)		*P* value
	2020 (n = 550)	2019 (n = 590)	
Sex			
Female	254 (46)	303 (51)	.09
Male	296 (54)	287 (49)	
Age, y			
Mean (SD)	70 (13)	72 (12)	.23
Median (IQR)	73 (24-96)	74 (26-99)	
Tumors, No.	567^a^	608^a^	
Location^b^			
Colon	408 (72)	429 (71)	.50
Rectum	158 (28)	179 (29)	
cT stage			
T1-T2	124 (22)	163 (27)	<.001
T3	189 (33)	252 (41)	
T4	172 (30)	132 (22)	
Tx	82 (14)	61 (10)	
cN stage			
N0	235 (41)	278 (46)	.02
N1-2	290 (51)	306 (50)	
Nx	42 (7.4)	24 (4.0)	
cM stage			
M0	447 (79)	473 (78)	.37
M1	117 (21)	127 (21)	
Mx	3 (0.53)	8 (1.3)	
Pretherapeutic multidisciplinary team assessment	523 (92)	560 (92)	.93
Planned for surgery	478 (84)	490 (81)	.10
Any surgical intervention the same year	439 (77)	444 (73)	.09

^a^ Eighteen patients had 2 tumors in 2019, and 17 patients had 2 tumors in 2020.

^b^ Location was not assessable in 1 patient in 2020.

### Colon Cancer

In the period March to August 2020, 396 patients received a diagnosis of colon cancer, of whom 319 (81%) underwent surgery the same year. In March to August 2019, 414 patients received a diagnosis of colon cancer and 315 (76%) underwent surgery the same year. Emergent resection surgery was equally common in 2020 and 2019 (13% [40 patients] vs 10% [32 patients]). Similarly, there were no differences in types of surgical procedures, with right-sided resection being the most common procedure both years. There was no delay in surgery during the pandemic, when the median (IQR) time from diagnosis to surgery was 29 (21-43) days in 2020, compared with 31 (23-42) days in 2019. There were no differences in median (IQR) length of stay (5.0 [3.0-7.0] vs 5.5 [3.0-8.0] days) or postoperative complications. The proportions of patients who experienced complications within 30 days from surgery were 11% (33 patients) in 2020 and 14% (43 patients) in 2019, and the corresponding numbers for ICU care were 4.6% (14 patients) vs 4.9% (15 patients), respectively. The total number of created ostomies almost doubled during the pandemic, from 55 in 2019 to 102 in 2020, and 30% of patients with colon cancer (96 patients) received an ostomy during the pandemic compared with 17% (53 patients) the year before (absolute risk, 13.0%; 95% CI, 6.8% to 20.0%). All types of ostomies were more common during the pandemic: the proportion of preoperative diverting stomas increased from 4.6% (14 patients) to 9.6% (29 patients) (absolute risk, 5%; 95% CI, 0.94% to 9.0%), temporary diversion at resection surgery increased from 8.1% (25 patients) to 13.0% (41 patients) (absolute risk, 5.3%; 95% CI, 0.43% to 10.0%) and permanent stoma increased from 4.9% (15 patients) to 12.0% (37 patients) (absolute risk, 7.3%; 95% CI, 2.9% to 12.0%). The highest surgical competence during surgery was unchanged, with a certified specialist in coloproctology according to the European Board of Surgical Qualification present in 63% of operations in 2020 (193 patients) vs 59% of operations in 2019 (180 patients), but the participation of resident surgeons decreased in 2020 from 35% (108 patients) to 27% (83 patients) (absolute risk difference, −7.90%; 95% CI, −15.00% to −0.55%) (Table 2).

**Table 2.** Surgical Treatment of Patients Who Received a Diagnosis of Colon Cancer in the Stockholm-Gotland Region, March 1 to August 31, 2020 and 2019

Variable	Patients, No. (%)		Absolute risk difference, % (95% CI)
	2020	2019	
Patients, No.	396	414	−18
Patients treated within the year of diagnosis	319 (81)	315 (76)	4.5 (−1.2 to 10)
Patients who received a stoma	96 (30)	53 (17)	13 (6.8 to 20)
Surgical treatments same year, No.	331	326	5
Stomas created	102 (31)	55 (17)	14 (7.5 to 20)
Treatment			
Polypectomy, local resection, or appendectomy	15 (4.5)	14 (4.3)	0.24 (−2.9 to 3.4)
Laparotomy or laparoscopy without resection	12 (3.6)	5 (1.5)	2.1 (−0.3 to 4.5)
Resection surgery	304 (92)	307 (94)	−2.3 (−6.2 to 1.6)
Type of resection surgery			
Anterior resection/abdominoperineal resection	25 (8.2)	22 (7.2)	1.1 (−3.2to 5.3)
Hartmann operation	9 (3.0)	4 (1.3)	1.7 (−0.63 to 4.0)
Sigmoid resection	59 (19)	50 (16)	3.1 (−3.0 to 9.2)
Left hemicolectomy	20 (6.6)	25 (8.1)	−1.6 (−5.7 to 2.6)
Transverse colon resection	2 (0.66)	1 (0.33)	0.33 (−0.78 to 1.4)
Colectomy	28 (9.2)	25 (8.1)	1.1 (−3.4 to 5.5)
Right-sided or ileocecal resection	161 (53)	180 (59)	−5.7 (−14 to 2.2)
Emergent resection surgery	40 (13)	32 (10)	2.7 (−2.4 to 7.8)
Time from diagnosis to surgery in elective surgery, median (IQR), d^a^	29 (21 to 43)	31 (23 to 42)	−3.4 (−9.0 to 2.2)
Diverting stoma preoperatively	29 (9.6)	14 (4.6)	5.0 (0.94 to 9.0)
Permanent stoma at resection surgery	37 (12)	15 (4.9)	7.3 (2.9 to 12)
Diverting stoma at resection surgery	41 (13)	25 (8.1)	5.3 (0.43 to 10)
Laparoscopic surgery	157 (52)	176 (57)	−5.7 (−14 to 2.2)
Resident physician present	83 (27)	108 (35)	−7.9 (−15 to −0.55)
Certified specialist in coloproctology	193 (63)	180 (59)	4.8 (−2.9 to 13)
Hospital stay, median (IQR), d^b^	5.0 (3.0 to 7.0)	5.5 (3.0 to 8.0)	−0.47 (−1.5 to 0.58)
Surgical complication within 30 d^c^	33 (11)	43 (14)	−3.2 (−8.4to 2.1)
Intensive care unit stay	14 (4.6)	15 (4.9)	−0.28 (−3.6 to 3.1)
Reoperation within 30 d	21 (6.9)	28 (9.1)	−2.2 (−6.5 to 2.1)
Death within 30 d	5 (1.6)	4 (1.3)	0.34 (−1.6 to 2.2)

^a^ Time was not assessable for 3 patients in 2019 and 4 patients in 2020.

^b^ Information was missing for 3 patients in 2019 and 5 patients in 2020.

^c^ Defined as a Clavien-Dindo score of 2 or higher.

At resection surgery, the probability of ostomy was significantly higher in 2020, with an absolute risk difference of 13.0% (95% CI, 6.4%-19.0%). The increase in ostomy use was largest among patients with distal tumors (16% [from 135 to 161 procedures]) and in patients with American Society of Anesthesiologists classification 3 to 4 (20%) (Table 3). An increase in stoma formation from 6.4% (3 patients) to 15.0% (13 patients) was seen also in the COVID-19–free Ersta hospital (eTable 1 and eTable 2 in the Supplement).

**Table 3.** Stomas in Patients With Colon Cancer Undergoing Resection Surgery for Colon Cancer in Stockholm-Gotland Region, March 1 to August 31, 2019 and 2020

Patient group	2020		2019		Absolute risk difference, % (95% CI)
	Stomas, No.	Procedures, No.	Stomas, No.	Procedures, No.	
All resection surgery	78	304	40	307	13 (6.4 to 19)
M0	62	278	23	281	14 (8.3 to 20)
Elective surgery	57	264	26	275	12 (6.1 to 18)
Proximal tumor^a^	24	143	16	172	7.5 (−0.03 to15)
Distal tumor^b^	54	161	24	135	16 (6.0 to 26)
American Society of Anaestesiologists score^c^					
1-2	28	170	15	158	7.0 (−0.23 to 14)
3-4	49	133	23	137	20 (9.7 to 30)

^a^ Defined as cecum to (and including) transverse colon.

^b^ Defined as from splenic flexure to distal sigmoid colon.

^c^ Information on American Society of Anaestesiologists classification was missing for 12 patients in 2019 and 1 patient in 2020.

### Rectal Cancer

For rectal cancer, the number patients who received a diagnosis was 158 in March to August 2020 and 178 in March to August 2019. There were no differences between 2020 and 2019 in the proportion of patients receiving preoperative radiotherapy (44% [46 patients] vs 45% [53 patients]) or preoperative chemotherapy (16% [17 patients] vs 18% [21 patients]). The median (IQR) time to surgery after neoadjuvant treatment was similar, 137 (113-162) days in 2020 and 146 (110-179) days in 2019. The corresponding numbers for patients treated with direct surgery were 35 (25-44 days and 37 (28-55) days from diagnosis, respectively. The most common surgical procedures were anterior resection (51% [44 patients] vs 46% [48 patients]) and abdominoperineal resection (38% [33 patients] vs 39% 41 patients]). The number of ostomies did not differ between the years (76% [81 patients] vs 80% [94 patients]). The length of stay, complications at 30 days, use of ICU care, and mortality were comparable between years (Table 4).

**Table 4.** Treatment of Rectal Cancer in the Stockholm-Gotland Region, March 1 to August 31, 2020 and 2019

Variable	Patients, No. (%)		Absolute risk difference, % (95% CI)
	2020	2019	
Patients with a diagnosis, No.	158	178	−20
Patients treated surgically within the year of diagnosis, No.	105 (67)	117 (66)	1.6 (−8.6 to 12)
Preoperative radiotherapy	46 (44)	53 (45)	−1.5 (−15 to 12)
Preoperative chemotherapy	17 (16)	21 (18)	−1.8 (−12 to 8.1)
Surgical treatments, No.	107	118	−11
Stomas created, No.	81 (76)	94 (80)	−4.0 (−15 to 6.9)
Treatment			
Local resection, transanal endoscopic microsurgery, or polypectomy	15 (14)	13 (11)	3.0 (−5.6 to 12)
Laparotomy or laparoscopy without resection	6 (5.6)	1 (0.85)	4.5 (0.10 to 9.4)
Resection surgery	86 (80)	104 (88)	−7.8 (−17 to 1.8)
Type of resection surgery			
Anterior resection	44 (51)	48 (46)	5.0 (−9.2 to 19)
Abdominoperineal resection	33 (38)	41 (39)	−1.0 (−15 to 13)
Sigmoid resection or Hartmann operation	8 (9.3)	14 (13)	−4.2 (−13 to 4.8)
Colectomy	1 (1.2)	1 (0.96)	0.20 (−2.7 to 3.1)
Time from diagnosis to surgery, median (IQR), d^a^			
With neoadjuvant treatment	137 (113 to 162)	146 (110 to 179)	−10 (−27 to 6.6)
Without neoadjuvant treatment	35 (25 to 44)	37 (28 to 55)	−2.7 (−12 to 6.3)
Diverting stoma preoperatively	14 (16)	13 (12)	3.8 (−6.3 to 14)
Permanent stoma at resection surgery	43 (50)	52 (50)	0 (−14 to 14)
Diverting stoma at resection surgery	31 (36)	40 (38)	−2.4 (−16 to11)
Laparoscopic surgery	64 (74)	78 (75)	−0.58 (−13 to 12)
Resident physician present	9 (10)	16 (15)	−4.9 (−14 to 4.6)
Certified specialist in coloproctology	79 (92)	86 (83)	9.2 (−0.12 to 18)
Hospital stay, median (IQR), d^a^	5.0 (4.0 to 7.0)	6.0 (4.0 to 8.0)	−1.5 (−3.6 to 0.68)
Surgical complication within 30 d	11(13)	21 (20)	−7.4 (−18 to 3.1)
Intensive care unit stay	6 (7.0)	4 (3.8)	3.1 (−3.4 to 9.7)
Reoperation	7 (8.1)	9 (8.6)	−0.51 (−8.4 to 7.4)
Death within 30 d	1 (1.2)	2 (1.9)	−0.76 (−4.2 to 2.7)

^a^ Information was missing for 1 patient in 2020.

### Number of Patients and Procedures During the Entire Years of 2020 and 2019

In 2020, 1226 patients received a diagnosis of colorectal cancer (colon cancer, 866 patients; rectal cancer, 360 patients) compared with 1219 patients in 2019 (colon cancer, 868 patients; rectal cancer, 351 patients). During both years, the number of patients receiving diagnoses was highest in September and October. The number of surgical procedures (including local resections, and nonresection laparotomy or laparoscopy) was 979 in 2020 and 952 in 2019, with similar monthly variation between the years (Figure).

**Figure.**
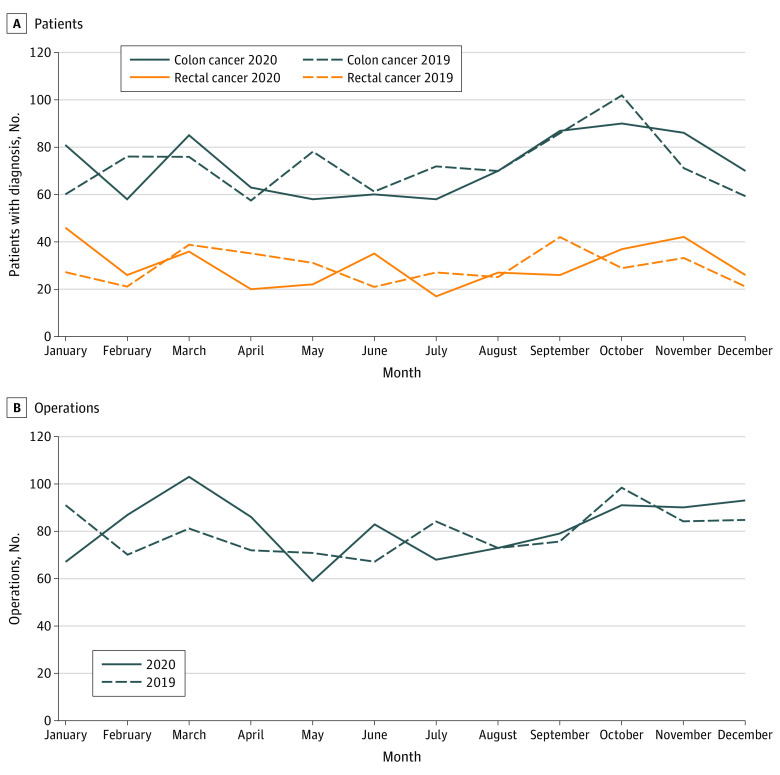
Number of Patients Receiving Diagnoses of Colon and Rectal Cancer and Number of Operations (Including Nonresection Surgery) per Month in the Stockholm-Gotland Region, 2020 and 2019

### Transfer of Patients

During the pandemic, it was more common to undergo surgery at another hospital than where the patient’s cancer had been diagnosed. South General hospital and St Göran hospital operated on fewer patients in March to August 2020 than in the same period 2019, and the COVID-19–free Ersta hospital operated on more patients (eFigure in the Supplement).

## Discussion

This cohort study found that during the first wave of the COVID-19 pandemic in 2020, when the Stockholm-Gotland region was heavily affected, slightly fewer patients received diagnoses of and treatment for colorectal cancer; however, during the entire year, the number of patients receiving diagnoses of and treatment for colorectal cancer was higher in 2020 than in 2019. Between March and August 2020, no increase in complications or in the proportion of emergency cancer resections was observed, nor was there any delay between diagnosis and operation for either colon or rectal cancer. The Stockholm area established a COVID-19–free hospital that continued to run a well-functioning service despite the pandemic.

No differences were observed in the number of laparoscopic surgical procedures, despite the lively debate worldwide on this subject.^15^ Recommendations from European Association of Endoscopic Surgery in April 2020 did not discourage the use of laparoscopic surgery, but recommended the use of personel protective equipment during all procedures to minimize the risk of virus spread from aerosol or body fluid.^16^ The most prominent difference during the first wave was the clear increase in ostomy formation, both temporary and permanent, in patients with colon cancer, probably reflecting efforts from surgeons to reduce complications to a minimum, as well as adherence to available guidelines.^8^

The subject of how colorectal cancer care has been affected by the COVID-19 pandemic has been an important issue worldwide.^4,17,18^ A study^19^ from Spain showed that 48% fewer patients received diagnoses of colorectal cancer during the first intense period of the pandemic compared with the year before, and that more patients received their diagnosis in an emergency setting. Two surveys from Italy reported a 62% reduction in colorectal cancer cases in 2020 compared with 2018,^20^ as well as a doubled waiting list.^21^ A British study^22^ reported a 63% reduction in monthly colorectal cancer cases in April 2020 compared with the same period 2019. By October 2020, the numbers were again comparable to those from the year before. These changes can be explained by postponed screening in many countries, including Sweden.^23^ The Swedish Cancer Registry has reported a 9% reduction in overall cancer cases, and an 11.8% reduction of colorectal cancer cases from March 2020 to October compared with the year before,^7^ which is a smaller reduction than many other countries have reported.

In the present study, there were 6.7% fewer diagnosed cases of colorectal cancer during the first wave of COVID-19 compared with the year before. However, the relative increase in T4 tumors and decrease in T1-3 tumors indicate that less-advanced tumors may be undiagnosed.

Many countries developed new recommendations for the management of colorectal cancer during the pandemic. In Italy, a cautious approach was recommended, with ostomies prioritized over anastomoses.^5^ In China, it was recommended to treat T3 and T4 tumors with neoadjuvant chemotherapy, to treat T1 tumors endoscopically, and to postpone surgery for T2 tumors.^24^ In Sweden, the temporary guidelines for the treatment of colorectal cancer aimed to maintain quality, to reduce the load on ICU care by minimizing complications, and to identify patients in need of urgent treatment. The first scenario recommended postponing treatment of premalignant conditions, and in some cases of advanced cancer, prolonging neoadjuvant treatment. A general recommendation was to choose the method of surgery with the lowest risk of complications (eg, ostomy instead of anastomosis) and to increase the use of temporary stomas (eg, loop stomas and Hartmann procedures). The present study indicates that Swedish surgeons changed their behavior in this direction. There was no increase in neoadjuvant oncological treatment, even though it was mentioned as a possibility in the national recommendations.^8^ Interestingly, ICU care, complications, and reoperations did not decrease, despite a changed surgical behavior with a much more frequent use of ostomies. This is in line with reports from Spain,^25^ where surgery was delayed, but complications and mortality were unchanged. Another international cohort study^26^ representing 40 countries showed higher mortality during the COVID-19 pandemic but fewer anastomotic leakages, perhaps indicating choice of safer surgical methods like stoma formation.

The current study found a significant decrease in residents’ participation in surgery, which has also been addressed previously.^27,28,29^ In a report from Chicago, Illinois,^30^ the authors described a strategy to optimize the situation for the residents, through maximizing learning occasions and support online teaching and learning. The decreased participation of residents in our setting can partly be explained by younger doctors being assigned to treat patients with COVID-19. It can also reflect an effort to shorten operation time and to reduce the number of personnel involved in patient care in the operating theater. We note that the presence of resident general surgeons in this type of surgery appears not to have been prioritized in the Stockholm region even in 2019, and the COVID-19 pandemic further accentuated this situation.

### Strengths and Limitations

The major strength of this study is its use of prospectively collected register data with high validity and nearly complete coverage. At the time of data collection, 97% to 98% of patients had complete registrations. In addition, the spread of the SARS-CoV-2 and subsequent load on the hospitals was greater in the Stockholm region and surroundings in this period, but later the same year the situation had deteriorated in other parts of Sweden, which makes the results of this study generalizable to a Swedish context.

Limitations include lack of information that are not included in the register (eg, routine blood tests such as albumin levels and hematocrit). We observed an increase in T4 tumors; therefore, it is possible that proportionally more patients presented with severe inflammatory response and, therefore, were considered at higher risk of surgical complications. We did not observe any increase in acute resection surgery, but more patients were treated with a 2-stage procedure with temporary diverting stoma before resection surgery. This may have been done as a strategy to delay resection surgery to a more safe, later stage.

We did not have information on COVID-19 positivity or whether the presence of an infection was associated with the choice of stoma formation. During the first period of COVID-19, mainly patients with symptoms were tested, and elective surgery was postponed in case of a positive result. In the COVID-19–free Ersta hospital, all patients were tested at admission. An increase in stoma formation in colon cancer surgery was also seen in the COVID-19–free hospital (eTable 1 in the Supplement). Given that the formation of stomas increased in all hospitals and in all types of colon cancer resection surgery (eTable 2 in the Supplement), we do not believe that COVID-19 positivity in patients can explain the general increase in stoma formation observed. It is more likely that increased stoma formation was associated with compliance with available surgical guidelines aiming to reduce perioperative morbidity during the pandemic.

Misclassification due to registration errors may have occurred but should be randomly distributed and not bias the results. Notably, despite a very good general coverage of the register, underreporting of anastomotic leakage has been observed in a Swedish study,^31^ and other complications may also be underreported. Globally, the complication rate for lower abdominal surgery has been reported to be 24.3%^32^ and in a Spanish Enhanced Recovery After Surgery (ERAS) setting 25%.^33^ However, underreporting bias should be the same between the years. Some differences observed between years may be due to natural variations.

## Conclusions

In this Swedish register-based cohort study of patients with colorectal cancer, in a time with high workload for the health care in the Stockholm region with numerous patients hospitalized because of COVID- 19, the number of patients with colorectal cancer treated, complications, and time to surgery remained unchanged. We saw a large increase in the ostomy formation in patients with colon cancer during the pandemic, and a lower participation of resident surgeons.

## Notes

Supplement. **eTable 1.** Surgical Treatment of Patients Diagnosed With Colon Cancer in the Stockholm-Gotland Region and in the COVID-19-Free Ersta Hospital March 1 to August 31, 2020 and 2019 **eTable 2.** Number of Procedures and Per-Operative Stomas in Colon Cancer Patients Undergoing Resection Surgery for Colon Cancer in Stockholm-Gotland Region and in the COVID-19-Free Ersta Hospital March 1 to August 31, 2019 and 2020 **eFigure.** Operating Hospital March 1 to August 31 in 2020 and 2019
