# Supplementary material for: Trends in Treatment of Colorectal Cancer and Short-term Outcomes During the First Wave of the COVID-19 Pandemic in Sweden
Source: JAMA Netw Open. 2022 May 9;5(5):e2211065. doi: 10.1001/jamanetworkopen.2022.11065 (PMC9086839; doi:10.1001/jamanetworkopen.2022.11065)
Supplement: Supplement. — eTable 1. Surgical Treatment of Patients Diagnosed With Colon Cancer in the Stockholm-Gotland Region and in the COVID-19-Free Ersta Hospital March 1 to August 31, 2020 and 2019 eTable 2. Number of Procedures and Per-Operative Stomas in Colon Cancer Patients Undergoing Resection Surgery for Colon Cancer in Stockholm-Gotland Region and in the COVID-19-Free Ersta Hospital March 1 to August 31, 2019 and 2020 eFigure. Operating Hospital March 1 to August 31 in 2020 and 2019 [file jamanetwopen-e2211065-s001.pdf]

Supplemental Online Content

Eklöv K, Nygren J, Bringman S, et al. Trends in treatment of colorectal cancer and short-term outcomes during the first wave of the COVID-19 pandemic in Sweden. *JAMA Netw Open*. 2022;5(5):e2211065. doi:10.1001/jamanetworkopen.2022.11065

eTable 1. Surgical Treatment of Patients Diagnosed With Colon Cancer in the Stockholm-Gotland Region and in the COVID-19-Free Ersta Hospital March 1 to August 31, 2020 and 2019
eTable 2. Number of Procedures and Per-Operative Stomas in Colon Cancer Patients Undergoing Resection Surgery for Colon Cancer in Stockholm-Gotland Region and in the COVID-19-Free Ersta Hospital March 1 to August 31, 2019 and 2020
eFigure. Operating Hospital March 1 to August 31 in 2020 and 2019

This supplemental material has been provided by the authors to give readers additional information about their work.

© 2022 Eklöv K et al. *JAMA Network Open.*

eTable 1. Surgical Treatment of Patients Diagnosed With Colon Cancer in the Stockholm-Gotland Region and in the COVID-19-Free Ersta Hospital March 1 to August 31, 2020 and 2019

	**Stockholm-Gotland region**			**Ersta hospital**		
	**2020**	**2019**	**Absolute risk difference 2019 to 2020 (95% CI)**	**2020**	**2019**	**Absolute risk difference 2019 to 2020 (95% CI)**
	N(%)	N(%)				
Number of diagnosed patients	396	414	-18	86	47	+39
Number of patients treated within the year of diagnosis	319 (81)	315 (76)	+4.5% (-1.2 to 10)	85 (99)	45 (96)	+3.1 (-3.1 to 9.3)
Number of patients that received a stoma	96 (30)	53 (17)	**+13% (6.8 to 20)**	13 (15)	3 (6.7)	+8.6 (-1.9 to 19)
Number of surgical treatments same year	331	326	+5	89	47	+42
Number of stomas created	102 (31)	55 (17)	**+14% (7.5% to 20%)**	13 (15)	3 (6.4)	+8.2 (-1.9 to18)
Treatment						
Polypectomy/local resection/appendectomy	15 (4.5)	14 (4.3)	+0.24% (-2.9% to 3.4%)	6 (6.7)	1 (2.1)	+4.6 (-2.0 to 11)
Laparotomy/laparoscopy without resection	12 (3.6)	5 (1.5)	+2.1% (-0.3% to 4.5%)	3 (3.4)	0 (0)	+3.4 (-0.4 to 7.1)
Resectional surgery	304 (92)	307 (94)	-2.3% (-6.2% to 1.6%)	80 (90)	46 (98)	**-8.0 (-15 to -0.48)**
Type of resection surgery						
Anterior resection/APR	25 (8.2)	22 (7.2)	+1.1% (-3.2% to 5.3%)	13 (16)	11 (24)	-7.7 (-22 to7.1)
Hartmann’s operation	9 (3.0)	4 (1.3)	+1.7% (-0.63% to 4.0%)	2 (2.5)	1 (2.2)	+0.3 (-5.1 to 5.8)
Sigmoid resection	59 (19)	50 (16)	+3.1% (-3.0% to 9.2%)	7 (8.8)	1 (2.2)	+6.6 (-0.91 to 14)

© 2022 Eklöv K et al. *JAMA Network Open.*

Left sided resection	20 (6.6)	25 (8.1)	-1.6% (-5.7% to 2.6%)	4 (5.0)	6 (13)	-8.0 (-19 to 2.8)
Transverse colon resection	2 (0.66)	1 (0.33)	+0.33% (-0.78% to 1.4%)	0 (0)	0 (0)	-
Colectomy	28 (9.2)	25 (8.1)	+1.1% (-3.4% to 5.5%)	7 (8.8)	0 (0)	**+8.8 (2.6 to 15)**
Right-sided/ileocaecal resection	161 (53)	180 (59)	-5.7% (-14% to 2.2%)	47 (59)	27 (59)	+0.05 (-18 to 18)
Emergent resection surgery	40 (13)	32 (10)	+2.7% (-2.4% to 7.8%)	0 (0)	0 (0)	-
Time from diagnosis to surgery in elective surgery[2]	29 (21-43)	31 (23-42)	+3.4 (-2.2 to 9.0)[4]	25 (19-36)	27 (20-36)	-2.8 (-13 to 7.5)[4]
Diverting stoma preoperatively	29 (9.6)	14 (4.6)	**+5.0% (0.94% to 9.0%)**	0 (0)	0 (0)	-
Permanent stoma at resection surgery	37 (12)	15 (4.9)	**+7.3% (2.9% to 12%)**	6 (7.5)	1 (2.2)	+5.3 (-1.8 to 12)
Diverting stoma at resection surgery	41 (13)	25 (8.1)	**+5.3 (0.43% to 10%)**	6 (7.5)	2 (4.4)	+3.2 (-5.1 to 11)
Laparoscopic surgery	157 (52)	176 (57)	-5.7% (-14% to 2.2%)	63 (79)	40 (87)	-8.2 (-2.1 to 5.0)
Resident physician present	83 (27)	108 (35)	**-7.9% (-15% to -0.55%)**	0 (0)	0 (0)	-
Certified specialist in coloproctology	193 (63)	180 (59)	+4.8% (-2.9% to 13%)	62 (78)	20 (43)	**+34 (17 to 51)**
Hospital stay (days)[1] median (IQR)	5.0 (3.0-7.0)	5.5 (3.0-8.0)	-0.47 (-1.5 to 0.58)[4]	4.0 (3.0 to 5.0)	5.0 (3.0 to 6.0)	+0.38 (-1.5 to 2.2)[4]
Surgical complication within 30 days[3]	33 (11)	43 (14)	-3.2% (-8.4% to 2.1%)	3 (3.7)	2 (4.3)	-0.60 (-7.8 to 6.6)
Intensive care unit stay	14 (4.6)	15 (4.9)	-0.28% (-3.6% to 3.1)	3 (3.8)	3 (6.5)	-2.8 (-11 to 5.5)
Reoperation within 30 days	21 (6.9)	28 (9.1)	-2.2% (-6.5% to 2.1%)	3 (3.8)	1 (2.2)	-1.6 (-7.5 to 4.4)
Death within 30 days	5 (1.6)	4 (1.3)	+0.34% (-1.6% to 2.2%)	0 (0)	0 (0)	-

[1] Missing information for 3 patients in 2019 and 5 patients in 2020
[2] Not assessable in 3 patients in 2019 and 4 patients in 2020
[3] Clavien-Dindo >2
[4] Mean difference by t-test

© 2022 Eklöv K et al. *JAMA Network Open.*

eTable 2. Number of Procedures and Per-Operative Stomas in Colon Cancer Patients Undergoing Resection Surgery for Colon Cancer in Stockholm-Gotland Region and in the COVID-19-Free Ersta Hospital March 1 to August 31, 2019 and 2020

	**Stockholm-Gotland region**			**Ersta hospital**		
**Patient group**	**N stomas / N procedures 2020**	**N stomas/N procedures 2019**	**Absolute risk difference 2019 to 2020 (95% CI)**	**N stomas / N procedures 2020**	**N stomas/N procedures 2019**	**Absolute risk difference 2019 to 2020 (95% CI)**
All resection surgery	78/304	40/307	**+13% (6.4 to 19)**	12/80	3/46	+8.5 (-2.1 to 19)
M0	62/278	23/281	**+14% (8.3 to 20)**	11/78	1/44	**+12 (+2.9 to 21)**
Elective surgery	57/264	26/275	**+12% (6.1 to 18)**	12/80	3/46	+8.5 (-2.1 to 19)
Proximal tumour[1]	24/143	16/172	+7.5% (-0.03 to15)	3/38	1/25	+3.9 (-7.6 to 15)
Distal tumour[2]	54/161	24/135	**+16% (6.0 to 26)**	9/42	2/21	+12 (-5.8 to 30)
ASA 1-2[3]	28/170	15/158	+7.0% (-0.23 to 14)	4/56	2/33	+1.1 (-9.5 to 12)
ASA 3-4[3]	49/133	23/137	**+20% (9.7 to 30)**	8/24	1/11	+24 (-1.1 to 50)

ASA, American Society of Anaestesiologist score

[1] Caecum to (and including) transverse colon

[2] From splenic flexure to distal sigmoid colon

[3] Information on ASA classification missing for 12 patients in 2019 and 1 patient in 2020

© 2022 Eklöv K et al. *JAMA Network Open.*

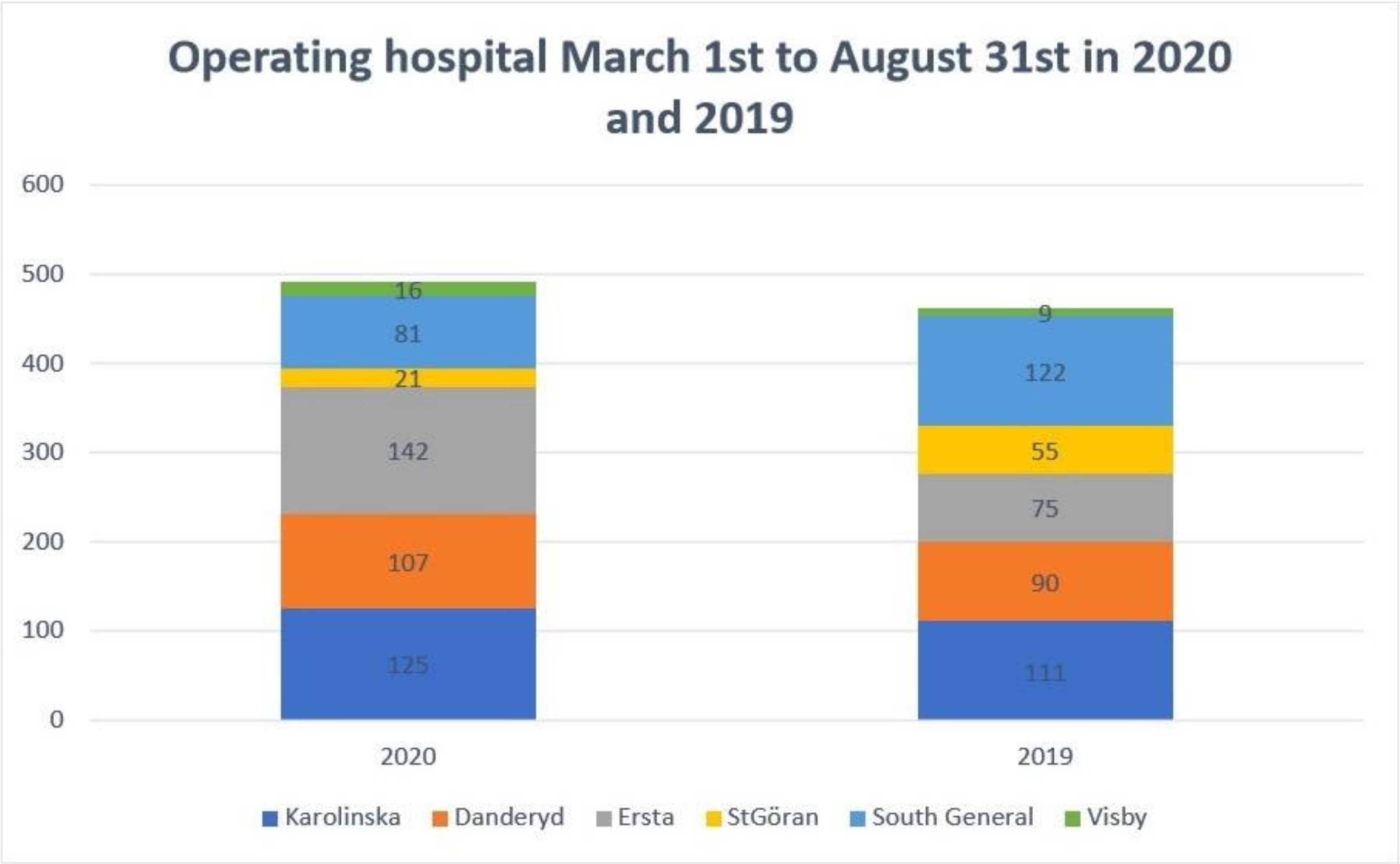

© 2022 Eklöv K et al. *JAMA Network Open.*
